# Supplementary material for: The unexplored diversity of rough-seeded lupins provides rich genomic resources and insights into lupin evolution
Source: Nat Commun. 2025 May 10;16:4358. doi: 10.1038/s41467-025-58531-w (PMC12065815; doi:10.1038/s41467-025-58531-w)
Supplement: Supplementary file 1 — Supplementary Information [file 41467_2025_58531_MOESM1_ESM.pdf]

The unexplored diversity of rough-seeded lupins provides rich genomic resources and insights into lupin evolution

Susek *et al.*

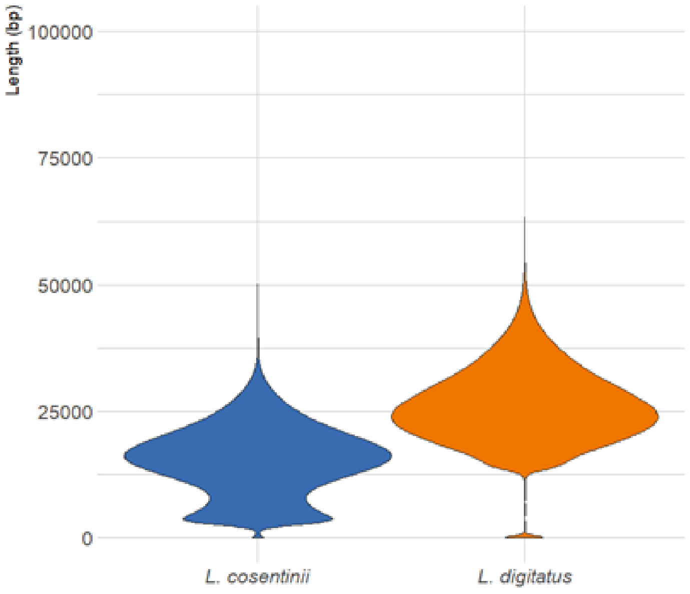

Supplementary Figure 1. Length distribution of PacBio sequencing reads for *Lupinus cosentinii* and *L. digitatus*. Source data are provided as a Source Data file.

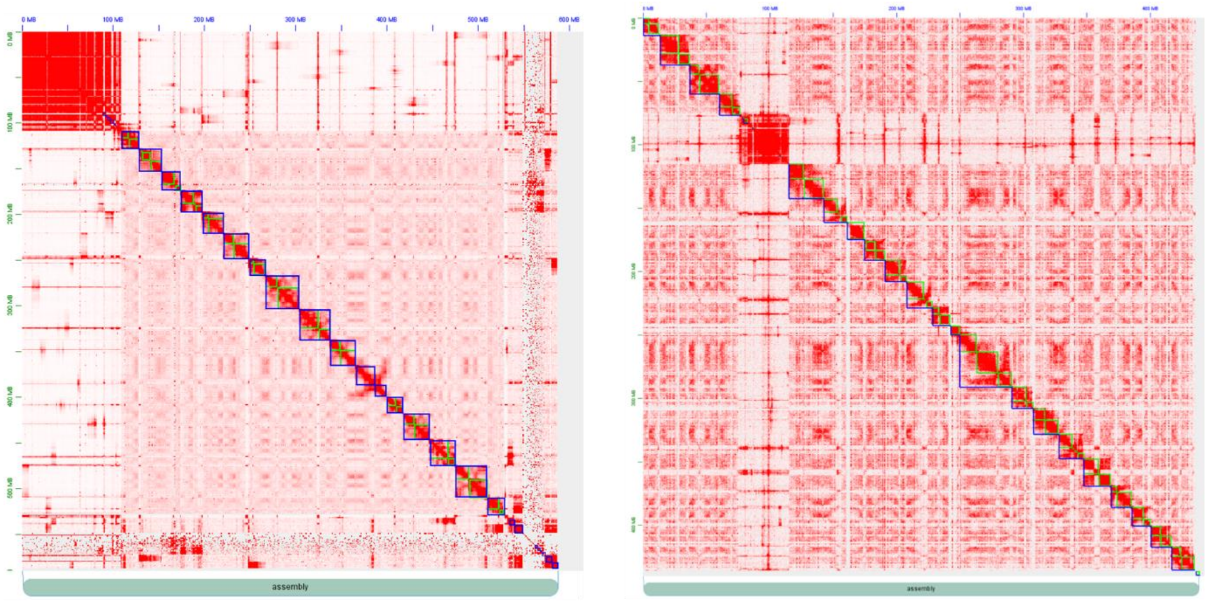

L.cosentinii L.digitatus

Supplementary Figure 2. Illumina Hi-C matrices of *Lupinus cosentinii* and *L. digitatus* genome assemblies.

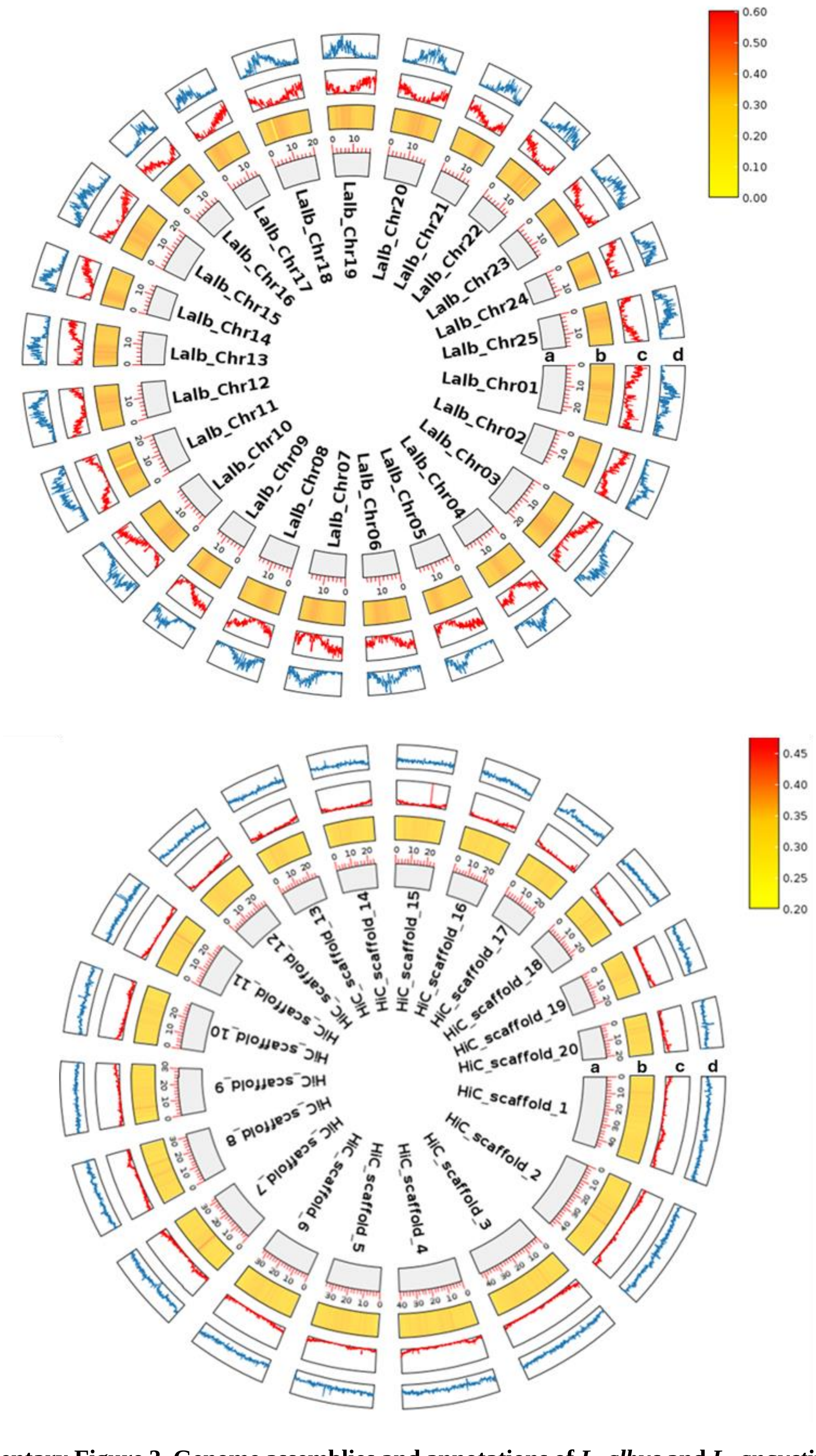

Supplementary Figure 3. Genome assemblies and annotations of *L. albus* and *L. angustifolius*. (a) Circular maps of *L. albus* (top) and *L. angustifolius* (bottom). (b) GC content of the genomes. (c) Gene density. (d) Repetitive element density. Source data are provided as a Source Data file.

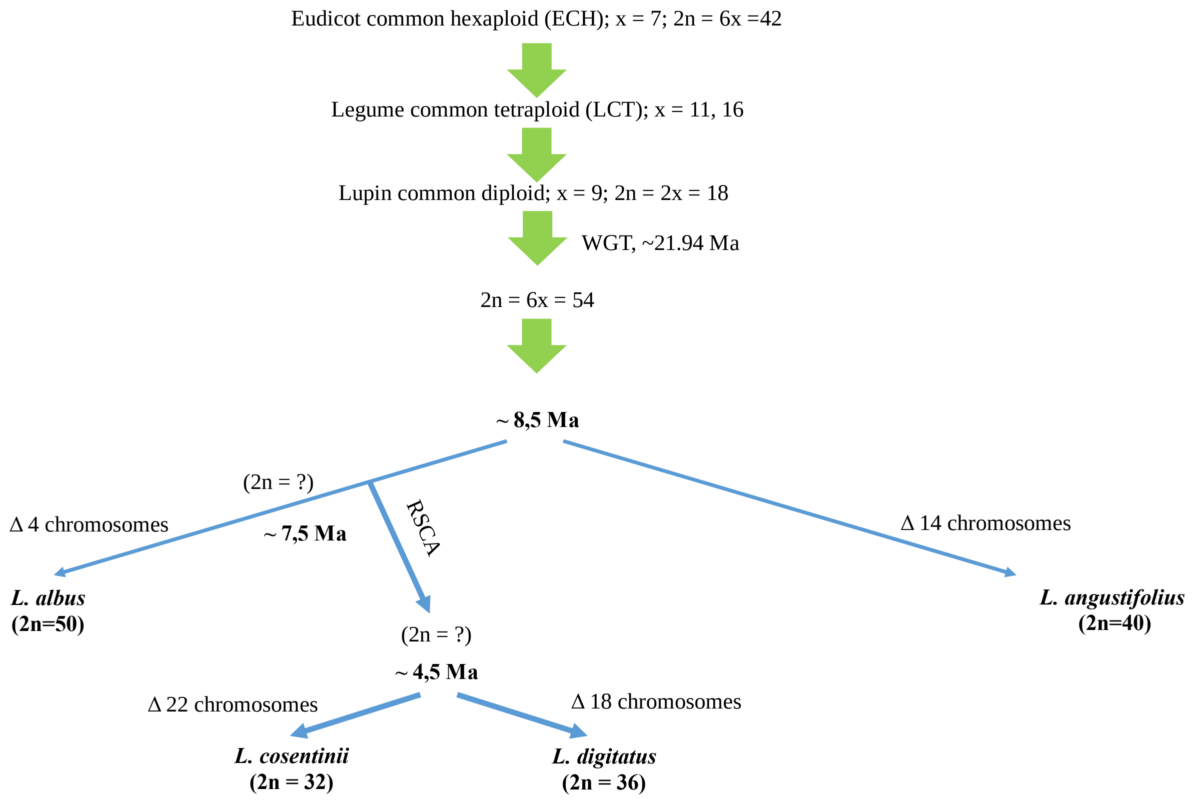

Supplementary Figure 4. Relationships among four lupin species with hypothetical scheme of karyotype evolution. RSCA – common ancestor of the Old World rough-seeded lupins; Δ – reduction of chromosome number relative to the WGT ancestral lupin 2n = 54. Divergence dates in million years ago (Ma) are based on Drummond *et al.*[1].

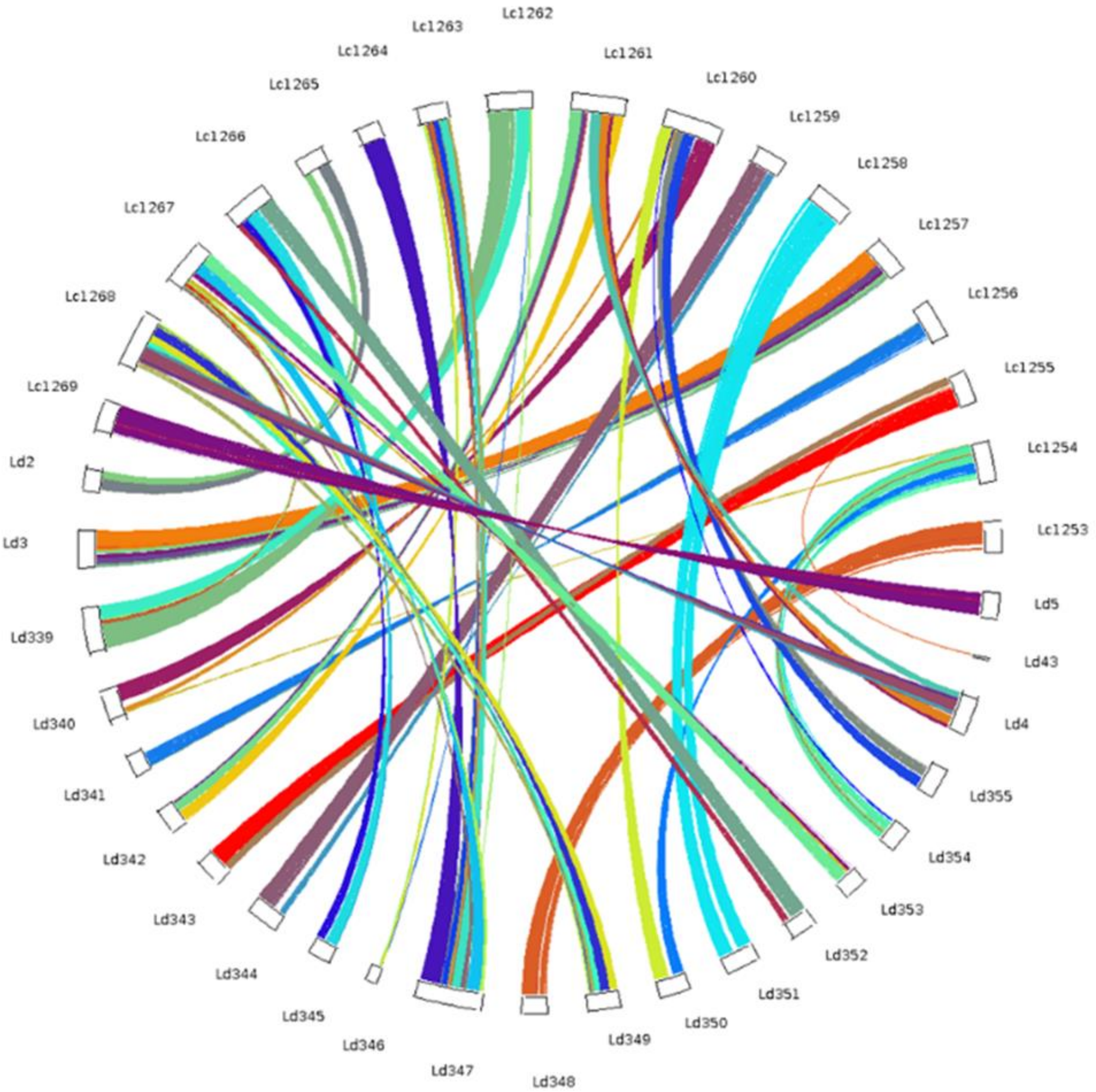

Supplementary Figure 5. Collinear blocks between *L. digitatus* (Ld) and *L. cosentinii* (Lc). Source data are provided as a Source Data file.

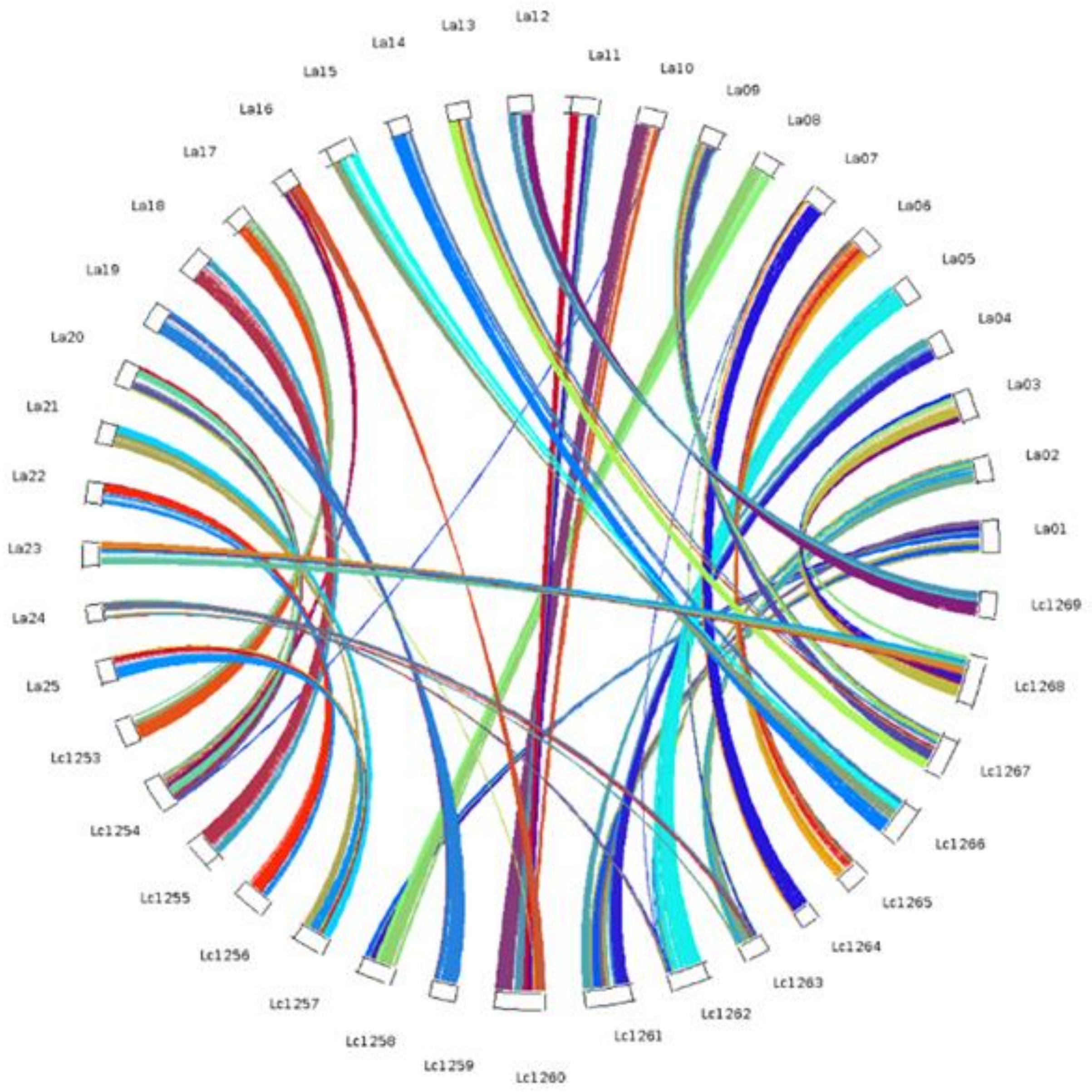

Supplementary Figure 6. Collinear blocks between *L. albus* (La) and L. cosentinii (Lc). Source data are provided as a Source Data file.

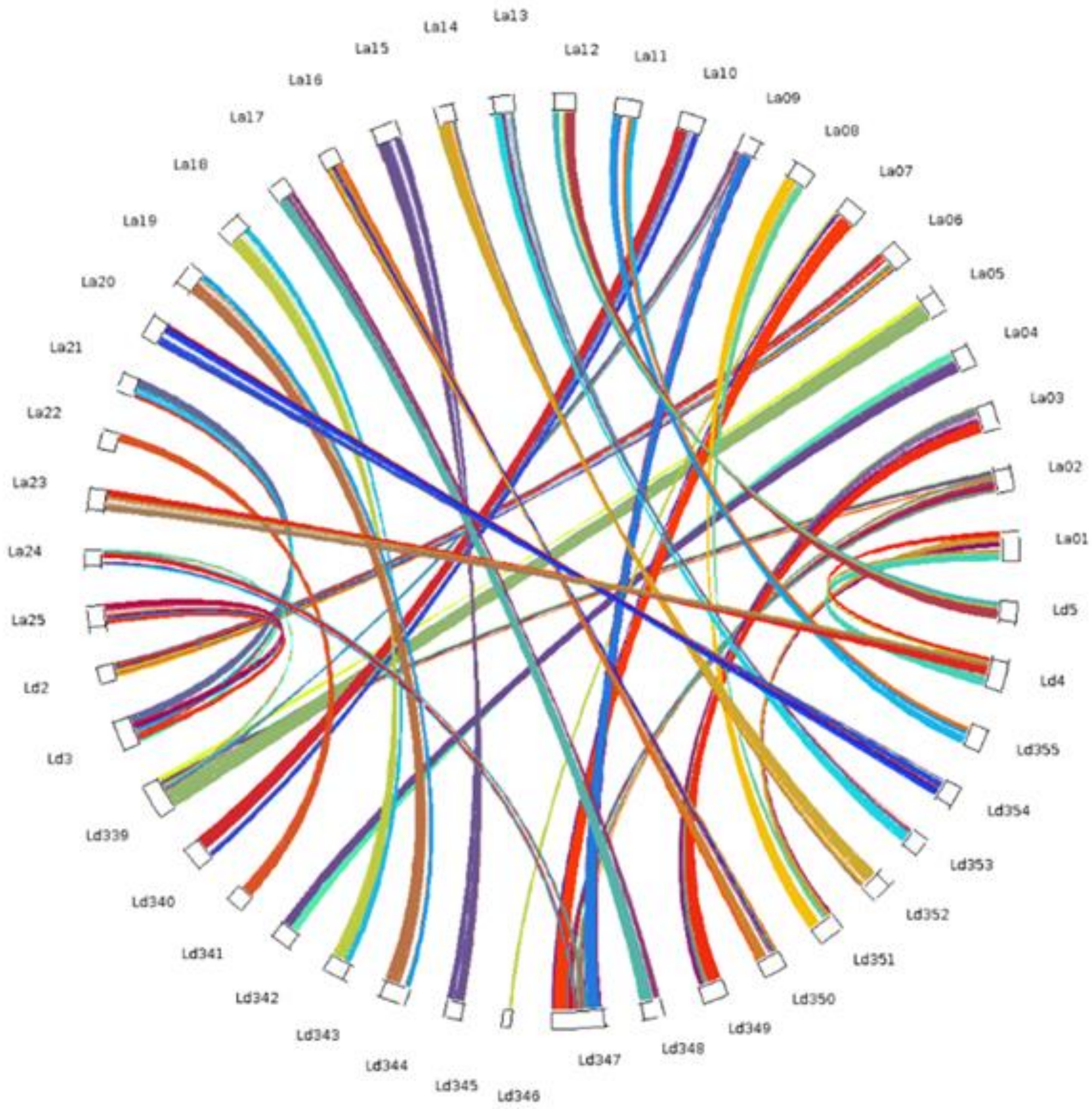

Supplementary Figure 7. Collinear blocks between *L. albus* (La) and *L. digitatus* (Ld). Source data are provided as a Source Data file.

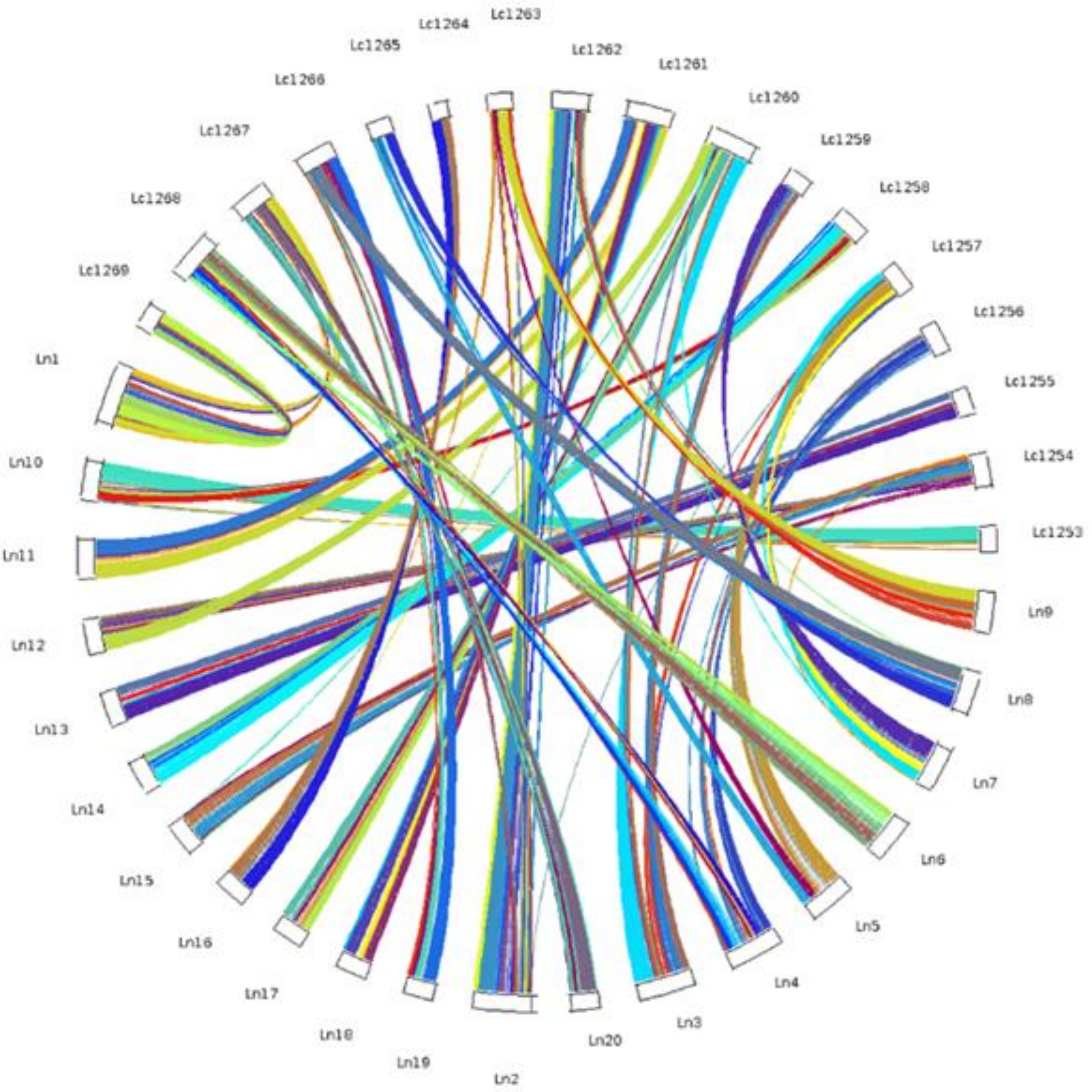

Supplementary Figure 8. Collinear blocks between *L. angustifolius* (Ln) and *L. cosentinii* (Lc). Source data are provided as a Source Data file.

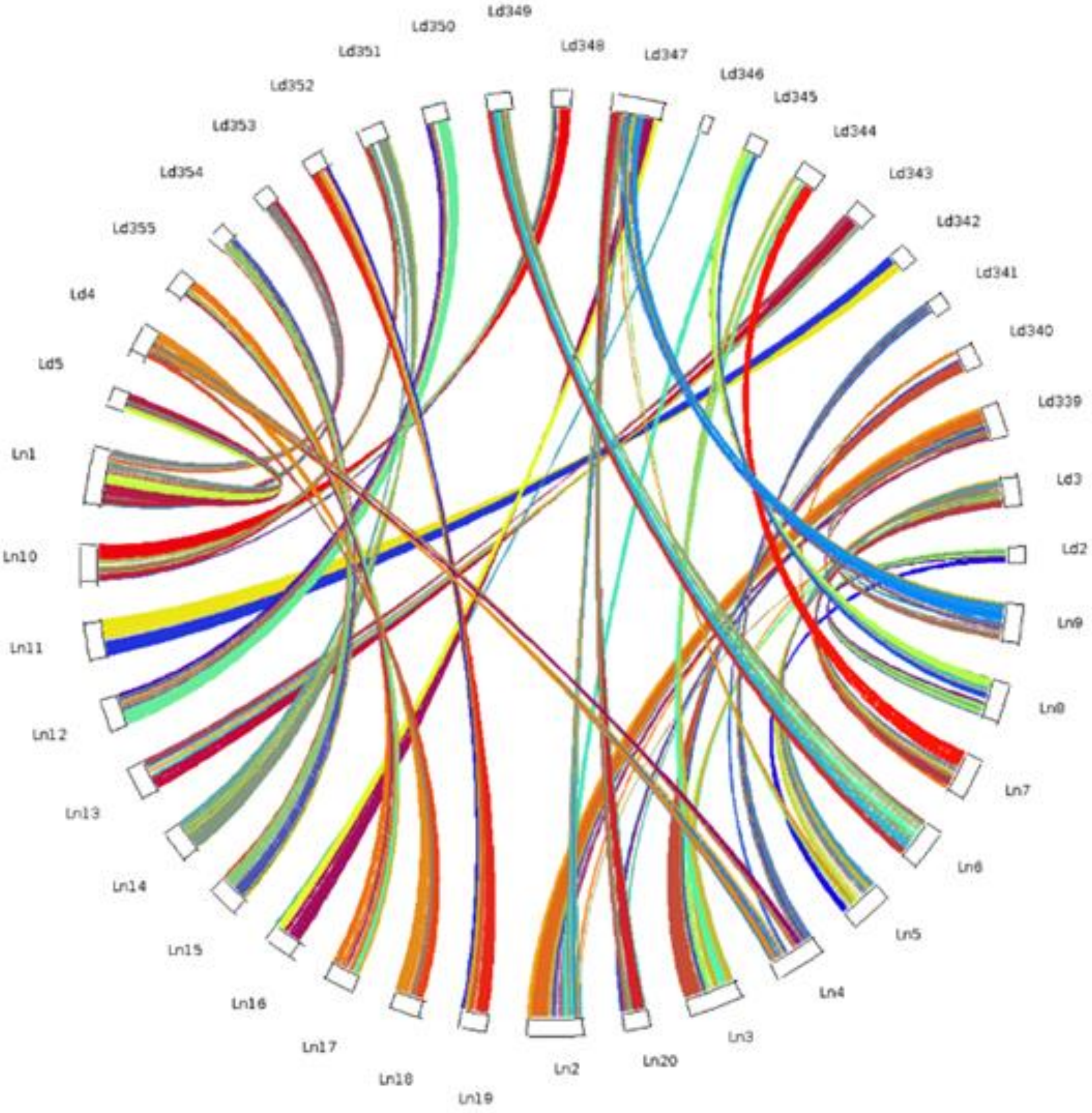

Supplementary Figure 9. Collinear blocks between *L. angustifolius* (Ln) and *L. digitatus* (Ld). Source data are provided as a Source Data file.

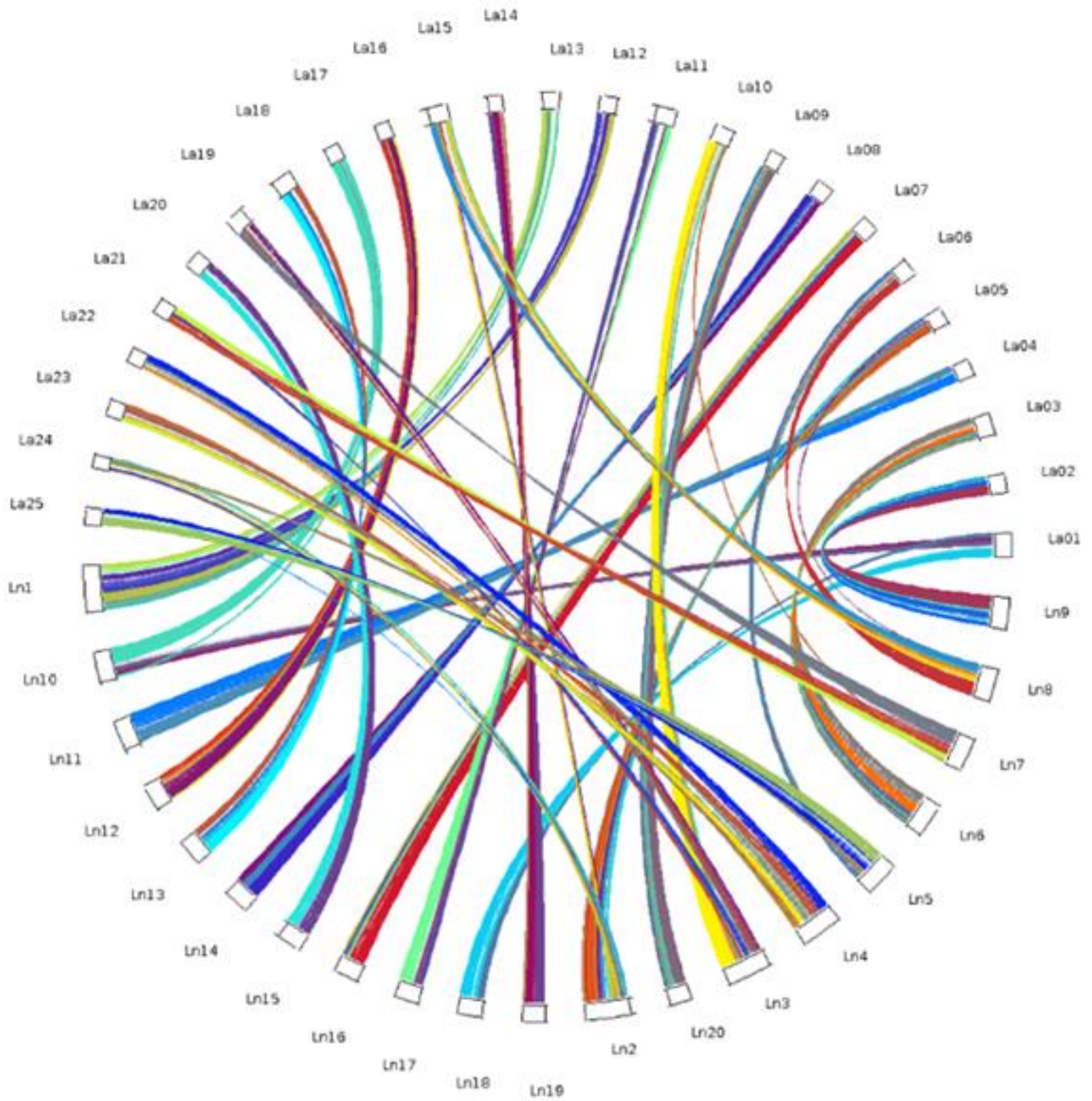

Supplementary Figure 10. Collinear blocks between *L. albus* (La) and *L. angustifolius* (Ln). Source data are provided as a Source Data file.

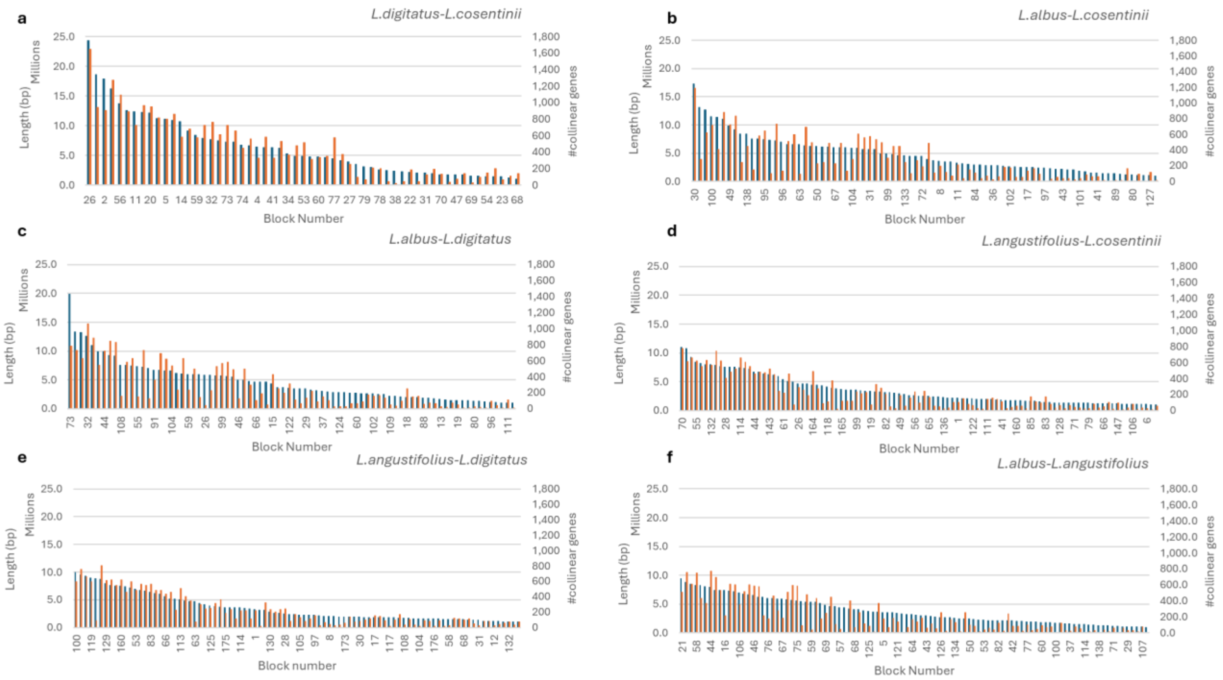

Supplementary Figure 11. Comparison of syntenic and collinear blocks. The graphs compare the length of syntenic blocks in Mbp (blue) on the left *y*-axis and the number of corresponding collinear blocks (orange) on the right *y*-axis. (a) Synteny between *L. digitatus* and *L. cosentinii*. (b) Synteny between *L. albus* and *L. cosentinii*. (c) Synteny between *L. albus* and *L. digitatus*. (d) Synteny between *L. angustifolius* and *L. cosentinii*. (e) Synteny between *L. angustifolius* and *L. digitatus*. (f) Synteny between *L. albus* and *L. angustifolius*. Source data are provided as a Source Data file.

Supplementary Table 1. Average degree of gene duplication in *L. cosentinii* and *L. digitatus* when considering the total number of genes and when focusing on genes in small syntenic blocks.

	L. cosentinii	***L. digitatus***
All genes	1.36	1.35
Genes in small syntenic blocks	1.43	1.43

Supplementary Table 2. Summary of expanded and contracted gene families, number of genes in families, and genes lost from *L. albus* and *L. angustifolius* as reference species.

		**Expanded**		**Contracted**	
		**Gene families**	**Genes**	**Gene families**	**Genes**
L. albus	*L. cosentinii*	2,751	3,268	4,071	5,546
	L. digitatus	2,784	3,370	4,075	5,571
L. angustifolius	*L. cosentinii*	1,413	1,903	3,680	6,021
	L. digitatus	1,618	2,208	3,862	6,249

Supplementary Table 3. Key characteristics of *L. cosentinii* and *L. digitatus*.

	L. cosentinii	***L. digitatus***
ID; provider	98460, doi:10.18730/1HA5M3; Polish *Lupinus* Collection (Poznan Plant Breeders Ltd., Wiatrowo branch, Poland)	PI 660697, doi:10.18730/1HA5N4; US Department of Agriculture, USA
Genome size (flow cytometry data, based on Plant DNA C-values database, Kew Royal Botanic Gardens)	695.80 (Mbp/1C)	671.30 (Mbp/1C)
Chromosome number	2n = 32	2n = 36
Geographic distribution	western Mediterranean coast	pan-Saharan region

Supplementary reference

[1] Drummond CS, Eastwood RJ, Miotto STS, Hughes CE. Multiple continental radiations and correlates of diversification in *Lupinus* (Leguminosae): Testing for key innovation with incomplete taxon sampling. *Syst Biol* **61**, 443-460 (2012).
