# Supplementary material for: The unexplored diversity of rough-seeded lupins provides rich genomic resources and insights into lupin evolution
Source: Nat Commun. 2025 May 10;16:4358. doi: 10.1038/s41467-025-58531-w (PMC12065815; doi:10.1038/s41467-025-58531-w)
Supplement: Supplementary file 2 — Description of Additional Supplementary Files [file 41467_2025_58531_MOESM2_ESM.pdf]

File Name: Supplementary Data 1
Description: Summary of the sequencing data obtained for *Lupinus cosentinii*, including WGS paired-end (PE), PacBio HiFi, Hi-C (PE), Bionano and RNA-Seq (PE).

File Name: Supplementary Data 2
Description: Summary of the sequencing data obtained for *Lupinus digitatus*, including WGS paired-end (PE), PacBio HiFi, Hi-C (PE), Bionano and RNA-Seq (PE).

File Name: Supplementary Data 3
Description: Statistics of the genome assemblies for *Lupinus cosentinii*: the first assembly made using HiCanu, after the polishing step with Pilon, after the purging procedure with purge_haplotigs, after the scaffolding with optical Bionano maps and, lastly, after scaffolding at the chromosome level with Hi-C data.

File Name: Supplementary Data 4
Description: Statistics of the genome assemblies for *Lupinus digitatus*: the first assembly made using HiCanu, after the polishing step with Pilon, after the purging procedure with purge_haplotigs, after the scaffolding with optical Bionano maps and, lastly, after scaffolding at the chromosome level with Hi-C data.

File Name: Supplementary Data 5
Description: Functional annotation in *Lupinus cosentinii*. This includes the three levels of confidence in annotation and the predicted Gene Ontology (GO) term.

File Name: Supplementary Data 6
Description: Functional annotation in *Lupinus digitatus*. This includes the three levels of confidence in annotation and the predicted Gene Ontology (GO) term.
